# Complete Genomic Sequence of the Clinical Isolate Legionella pneumophila Serogroup 1 Strain 80-045 from Japan

**DOI:** 10.1128/MRA.00822-21

**Published:** 2021-11-04

**Authors:** Masatomo Morita, Nanno Harada, Yuna Shinohara, Miyo Murai, Naoko Ishii, Junko Amemura-Maekawa, Yukihiro Akeda

**Affiliations:** a Department of Bacteriology I, National Institute of Infectious Diseases, Tokyo, Japan; b Department of Health Sciences, Saitama Prefectural University, Saitama, Japan; SIPBS, University of Strathclyde

## Abstract

We report the complete genomic sequence of Legionella pneumophila serogroup 1 strain 80-045, isolated from autopsy lung tissue of the first patient diagnosed with Legionnaires’ disease in Japan.

## ANNOUNCEMENT

Legionella pneumophila inhabits natural and human-made water environments as a symbiotic organism in free-living amebae (1). L. pneumophila was identified as the human opportunistic pathogen in an outbreak of fatal pneumonia at an American Legion convention in Philadelphia in 1976 (2). The first genomic sequence of Philadelphia 1, which was the isolate responsible for the outbreak, was reported as belonging to L. pneumophila (3). The first case of Legionnaires’ disease in Japan was reported as a fatal pneumonia case in 1980. The patient was a 64-year-old male with diabetes mellitus. The lung aspirates and lung tissue were inoculated onto buffered charcoal-yeast-extract agar plates; the colonies obtained were grayish-white and smooth, characteristic of *Legionella* spp., and were identified as L. pneumophila serogroup 1 using the direct fluorescent antibody method (4). Here, we report the complete genomic sequence of L. pneumophila strain 80-045.

The L. pneumophila isolate was grown on buffered charcoal-yeast-extract agar at 35°C for 90 h. Genomic DNA was extracted using a DNeasy blood and tissue kit (Qiagen, Hilden, Germany). Whole-genome sequencing was performed using the MiSeq (Illumina, San Diego, CA) and MinION (Oxford Nanopore Technologies, Oxford, UK) platforms. A genomic library was prepared using a Nextera XT DNA library prep kit and sequenced using the MiSeq platform. The raw sequence reads were filtered to remove low-quality sequences and trimmed to remove adaptor sequences using MiSeq Control Software 2.6.2.1. A genomic library was also prepared using a rapid sequencing kit (SQK-RAD004), and the library was sequenced on a MinION sequencer using an R9.4.1 flow cell (5). The raw data were base called using Guppy 5.0.11, and adaptors were removed before assembly using Porechop 0.2.4 (https://github.com/rrwick/Porechop) (6, 7). As a result, 952,592 short reads (total read length, 233,150,299 bp) and 13,657 long reads (total read length, 78,646,486 bp; maximum read length, 62,146 bp) were used for genome sequence assembly. A hybrid genome assembly was performed using Unicycler v0.4.8 in conservative mode (8). Annotation was performed using the DDBJ Fast Annotation and Submission Tool (9). Default parameters were used for all software programs unless otherwise specified.

The L. pneumophila strain 80-045 genome consisted of a 3,438,257-bp circular chromosome with 38.2% G+C content; it had 2,994 predicted coding sequences, 9 rRNAs, 43 tRNAs, 2 CRISPR arrays, and genes for the Dot/Icm type-IVB secretion system. Based on phylogenetic analysis of the 16S rRNA (10) and *mip* genes (11), strain 80-045 belonged to Legionella pneumophila. Sequence-based typing indicated that the strain belonged to sequence type 118 (ST118), which has been observed only in Japan (12). Therefore, the genomic information uncovered in this study will be a useful reference for L. pneumophila serogroup 1 in Japan.

### Data availability.

The genomic sequence of the L. pneumophila isolate, 80-045, was deposited at DDBJ/ENA/GenBank under accession number AP024961. Additionally, the short and long reads were deposited at the DDBJ/ENA/GenBank Sequence Read Archive under accession numbers DRR310440 and DRR310441, respectively.
